# Supplementary material for: Performance Evaluation of Particulate Matter and Indoor Microclimate Monitors in University Classrooms under COVID-19 Restrictions
Source: Int J Environ Res Public Health. 2021 Jul 9;18(14):7363. doi: 10.3390/ijerph18147363 (PMC8305222; doi:10.3390/ijerph18147363)
Supplement: Supplementary file 1 [file ijerph-18-07363-s001.zip › ijerph-1277358-supplementary.pdf]

Supplementary

Performance evaluation of particulate matter and indoor microclimate monitors in university classrooms under COVID-19 restrictions

Laurentiu Predescu[1] and Daniel Dunea[2,*]

[1] Department of Food Engineering, Faculty of Environmental Engineering and Food Science, Valahia University of Targoviste, Aleea Sinaia no.13, 130004 Targoviste, jud. Dambovita, Romania;
laurentiu.predescu@valahia.ro

[2] Department of Environmental Engineering, Faculty of Environmental Engineering and Food Science, Valahia University of Targoviste, Aleea Sinaia no.13, 130004 Targoviste, jud. Dambovita, Romania;
dan.dunea@valahia.ro

* Correspondence: dan.dunea@valahia.ro (D.D.); Tel.: +40-769-076-854

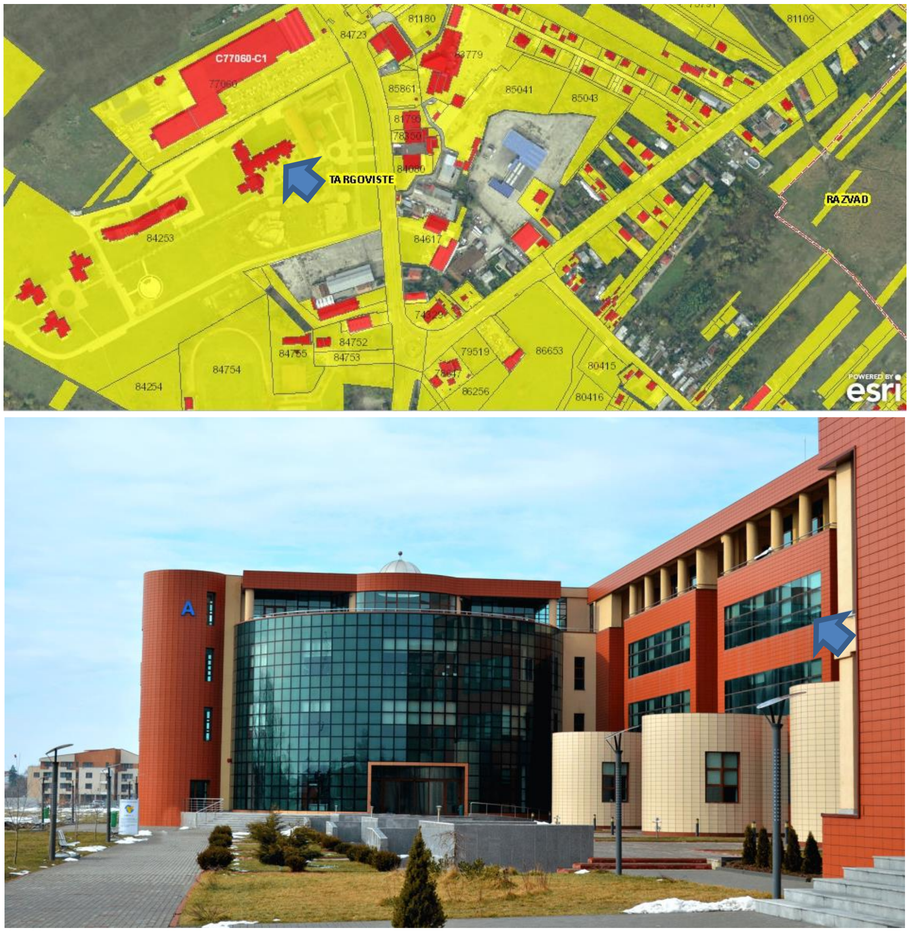

Fig. S1 Topographic map and image of Valahia University Campus showing the building in which the classroom is located at the 2nd floor (blue arrows) - http://geoportal.ancpi.ro/geoportal/imobile/Harta.html

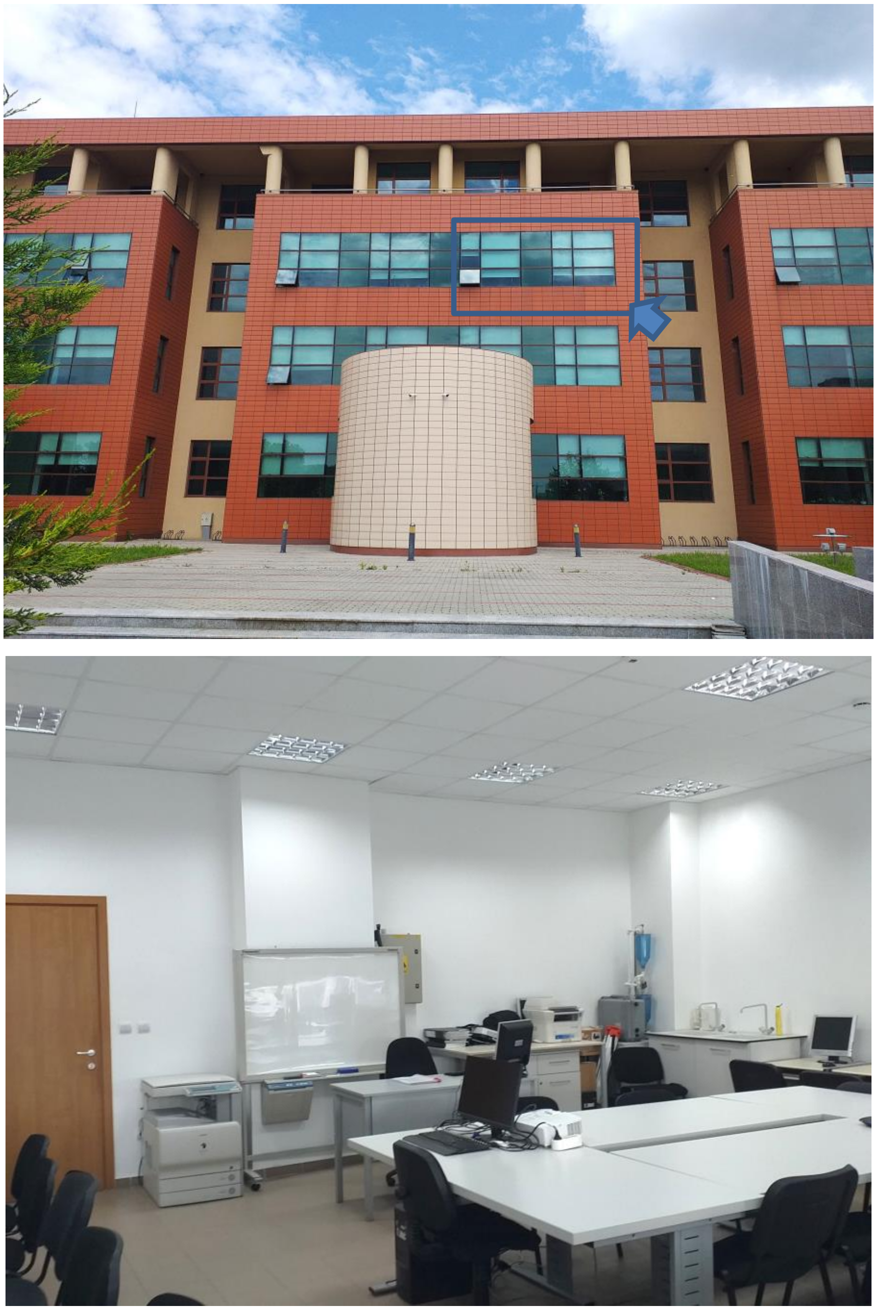

Fig. S2 Interior of the classroom located at the 2nd floor where the experiments have been performed.

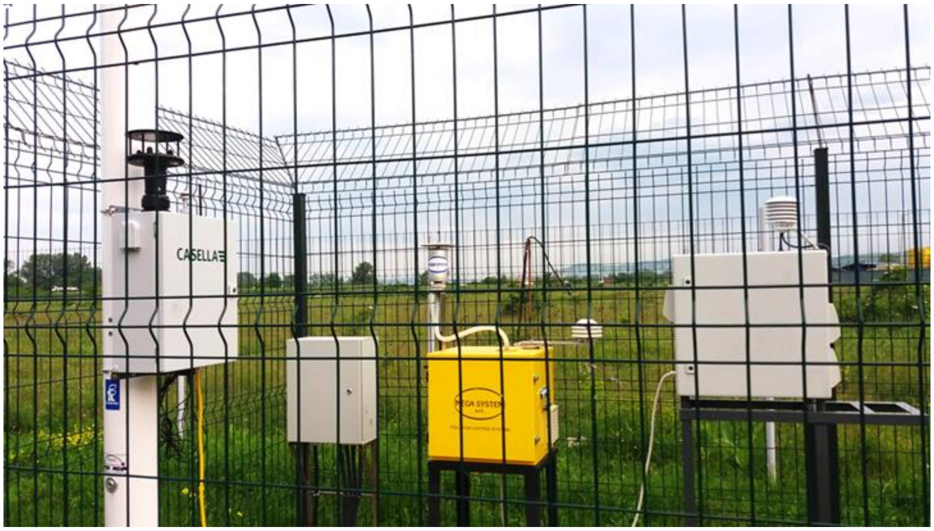

Fig. S3 Monitoring platform for particulate matter and meteorological parameters in Valahia University Campus (Casella Guardian 2, SELECT ONE Gravimetric Sampler, Rokidair microstation for PM measurements)

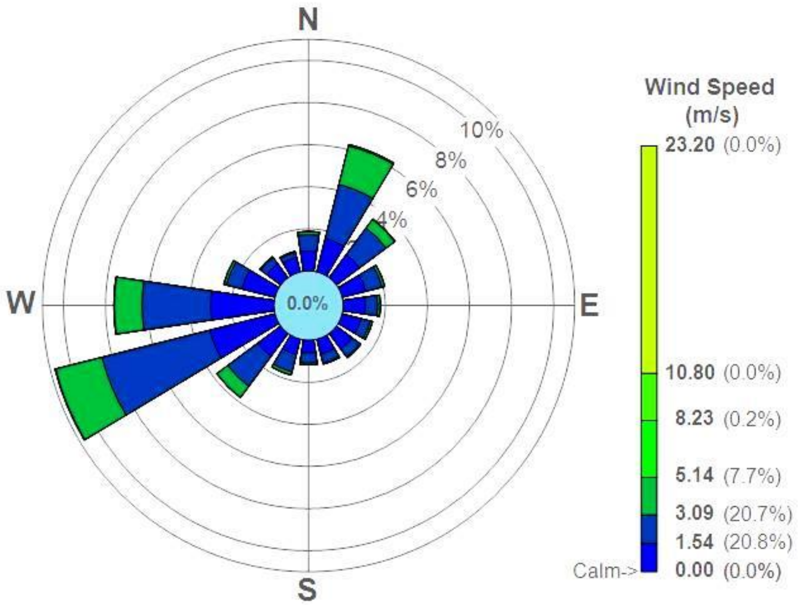

Fig. S4 Wind rose showing how wind speed and direction are typically distributed at the location (m s^{-1})

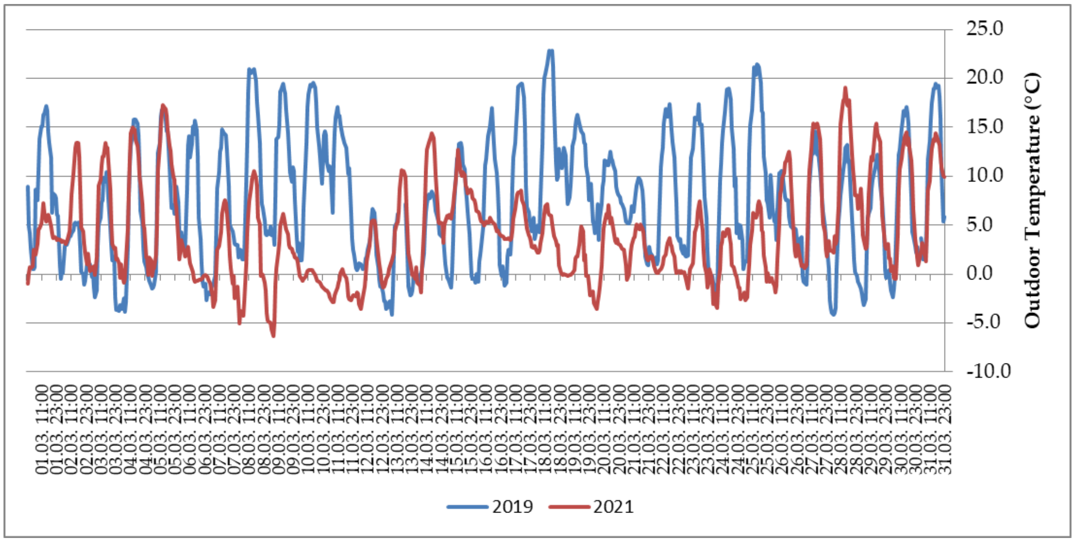

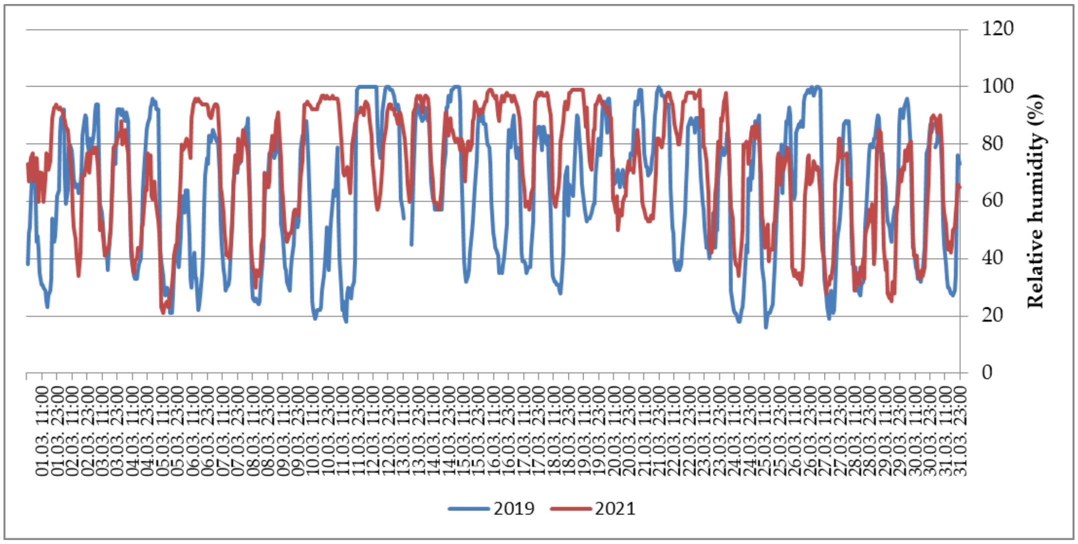

Fig. S5 Outdoor temperature (°C) and relative humidity (%) in March 2019 and 2021

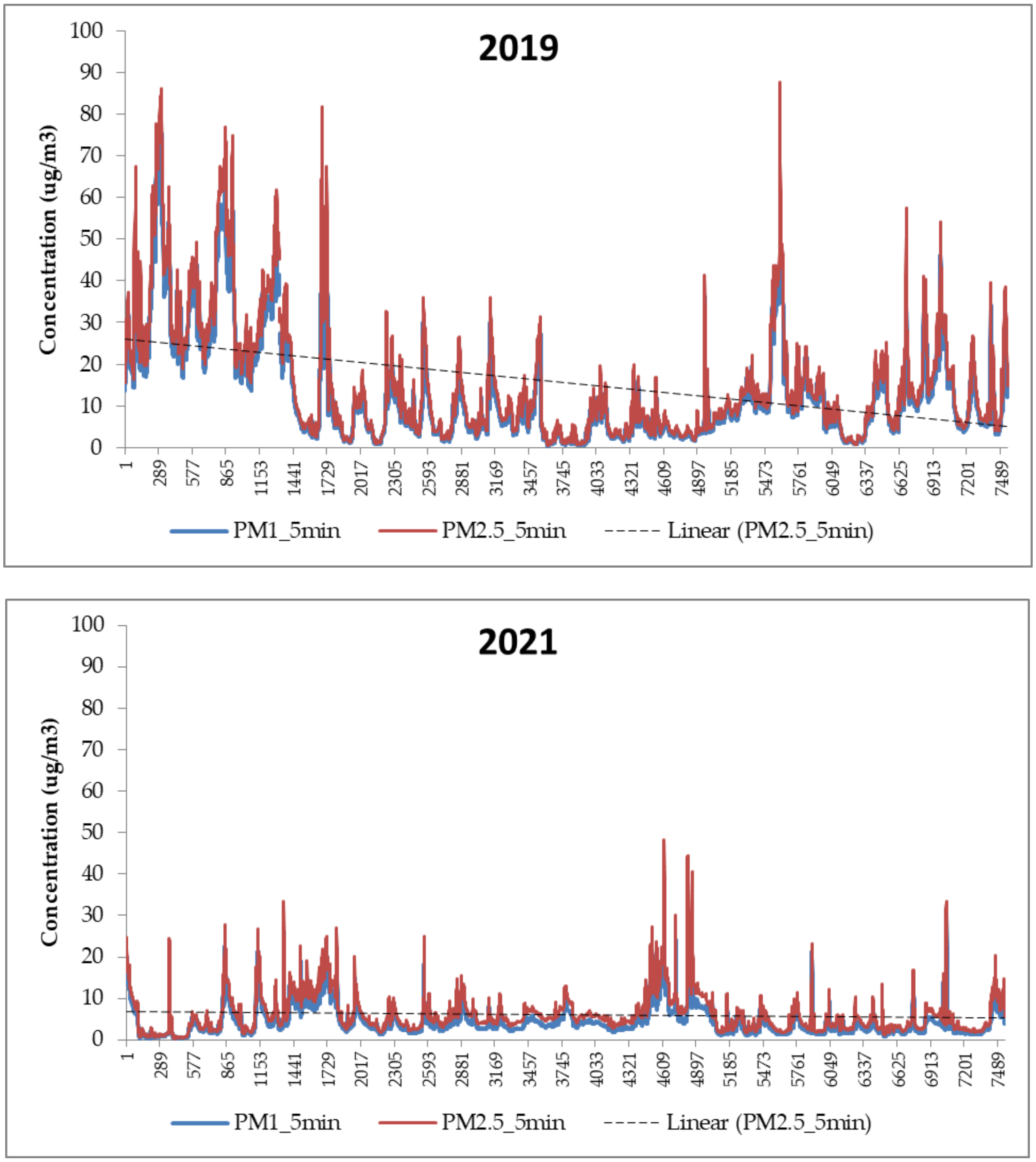

Fig. S6 Outdoor concentrations of $PM_{2.5}$ and PM_1 (μg m^{-3}) recorded at 5 minutes rate in March 2019 and 2021, respectively, using the optical monitors located in the Valahia University Campus (fig. S3)

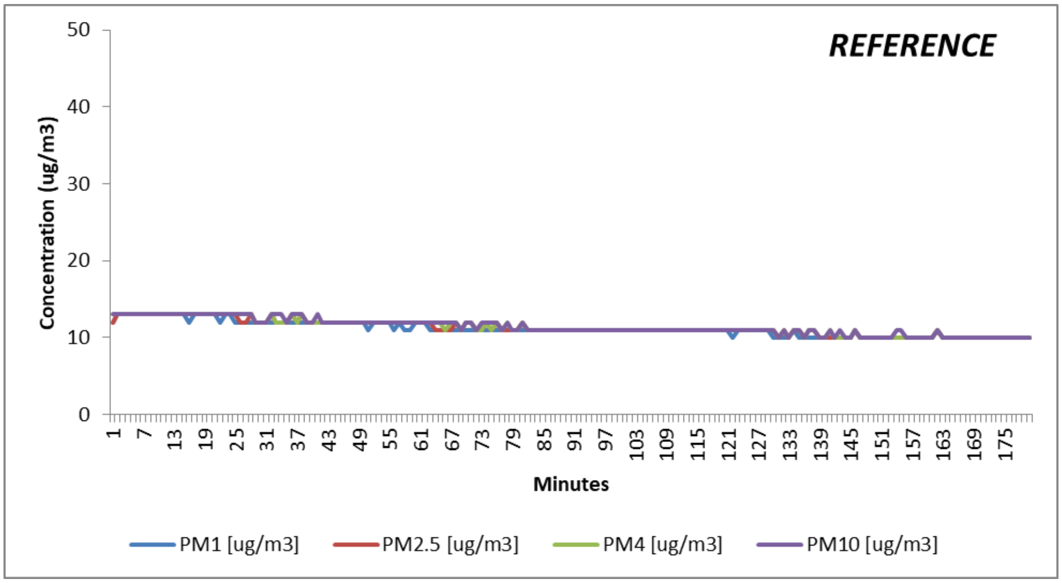

Fig. S7 Reference indoor concentration of particulate matter fractions in the classroom without students and with windows and door closed (3 hours time series)
